# Cases of Hydrothorax

**Published:** 1810-05

**Authors:** 


### *Cases of Hydrothorax*, by Dr. Maclean.*

I Had hitherto prescribed the digitalis only in an irregular and casual way, and my success was not such as to encourage farther trials. But often observing no sensible effect

---

* The analysis of Dr. Maclean's Work on hydrothorax, given by our Reviewer, in the last Number, is so concise, that we think it necessary, in justice to the merit of the Author, as well as to enable our Readers to form a judgment of the propriety and success of his mode of treating this formidable disease, to lay before them two or three of his cases. Ed.

fect either good or bad from what I had reason to believe were full doses of it, I began to suspect the quality of the preparations that were used. Accordingly, about this time I took some pains to procure the herb genuine, and began to cultivate it in my own garden. The result I have every reason to be satisfied with; indeed it has exceeded my most sanguine expectations. The fortunate issue of the following case, the first that occurred afterwards, induced me to pay still further attention to its preparation and exhibition.

CASE XII.

March 12, 1796.—Mrs. E. æt. 40, the mother of several children; of a constitution naturally delicate, and much weakened by the cares and fatigue of a large family. Has occasionally consulted me for three years back, for complaints which indicated great weakness and irritability of the heart and lungs, having been subject to quick, hurried, and difficult respiration, attended frequently with palpitation, or *fluttering*, as she termed it, of the heart, increased on bodily exertion or emotion of mind. Of this date I find the following memorandum in my case-book: "Symptoms of hydrops pectoris." A blister was directed to be applied to the chest, and some expectorant and diuretic medicines were prescribed, which soon removed her complaints.

June 23, 1798.—I find her again on my report book, with the same symptoms, for which similar remedies were prescribed.

July 13.—No relief from the last medicines. In every respect worse. Respiration very much interrupted, especially on motion, or on lying down in bed. Pulse quick, feeble, and extremely irregular; urine very small in quantity, and high coloured, with sediment; ankles and legs œdematous. Supposes herself in the fifth month of pregnancy; and there is every reason to suspect the fœtus has been dead for a fortnight, in which period her complaints have made very rapid progress. Has for some months suffered great mental anxiety and uneasiness, from causes unnecessary to state here; to which her present illness is to be attributed.

℞. Dig. purp. fol. nostr. pulv. gr. j. Confect. arom. q. s. ft. pilul. nocte maneq. cum mistur. infra præs. coch. No. ij. s.

℞. Fol. dig. purp. incis. ʒj. Infunde in aq. ferv. ℥viij. per hor. iv. dein cola.

 ℞. Infus

℞. Infus. colat, ℥v. Spt. juniper c. ℥ß. Spt. æth. nitr. Spt. æth. vitr. c. sing. ʒij. M.

Particular injunctions were given to discontinue the Foxglove as soon as it produced any of its unpleasant effects.

July 17.—I heard no more of her till the evening of this date, when my immediate attendance was desired. I found her retching violently, and bringing up at times a dark coloured matter, like coffee grounds; the extremities cold; the pulse extremely small, fluttering, and irregular; had frequent faintings, or, to use the language of those about her, "they could not keep life in her;" and the body was covered with cold sweats. She had been in this state for several hours: the cause of these alarming symptoms was sufficiently obvious. On inquiry, I found she had experienced immediate relief from the medicines; that the quantity of urine was very much increased; the swellings nearly gone, and that she was considered as mending very rapidly till this morning, when the stomach began to be disordered. I found also that she had been under the influence of the Foxglove nearly two days; yet, in direct contradiction to the directions rigidly enjoined, she was desired to continue it this morning! it was in vain that relief was attempted through the medium of the stomach; for every thing liquid, even in the smallest quantity, was immediately rejected. By an anodyne glyster, warm anodyne embrocations rubbed into the epigastrium, abdomen, and feet, small pills of solid opium with confect. aromat. all the urgent symptoms subsided in a few hours, and I had the pleasure of leaving my patient in the morning quiet and composed.

She continued gradually to mend from this time till about a fortnight after, when she miscarried of a fœtus which bore evident marks of having been dead at the time she apprehended. The water now began to accumulate in the chest and legs with unusual rapidity, and with it all her former distressing symptoms returned.—But although her strength was nearly exhausted, the water was speedily evacuated by the same means; and by a combination of tonics with diuretics, she was restored to health in a few weeks.—She has had no return of the dropsical symptoms, but has had occasional attacks of her former complaints, and always received advantage from medicine. On the whole, her health has been of late years improved.

In every point of view this case will appear interesting; an amiable woman has been rescued from a very alarming state, state, by a remedy which, by the most flagrant inattention, had nearly proved her destruction.*

The result of this case impressed on my mind so favorable an opinion of the foxglove, that I determined to direct my attention more than ever to it: from this period it has always formed a part of my remedy in dropsy; and my failures hitherto having been as much owing to the quality of the herb used, as to my ignorance of the proper dose and mode of exhibition, I furnished the practitioner with whom it was prescribed with a portion of the leaves out of my own garden. Having been absent from Britain when Withering's excellent work was published, and for some years afterwards, I had not the good fortune of seeing it at this time, and of borrowing light from so safe a guide.

CASE XXXIV.

March 30.—Mr. Partridge, of Bures, ætat. 49. nephew of the subject of case 17. Extreme difficulty in respiration, increased on the least motion, or on talking, attended for the most part with a peculiar wheezing noise. Has great tightness or sense of stricture and oppression across the chest. P. irregular, feeble, and frequent; it may be called a quick succession of weak irregular vibrations. Urine very scanty, high coloured, with a copious pink coloured sediment. Has been obliged to sleep in a chair for a fortnight, in consequence of which his legs, especially the left, are much swelled, and pitting on pressure. A small degree of tumefaction of the abdomen also. Whole face, especially the lips and nose, of a livid hue. Eyes dull, heavy, and expressive of great suffering; the conjunctiva somewhat yellow. Very little thirst. Appetite much impaired, as is his general strength. Was taken ill three weeks since with nearly all these symptoms, which have gradually increased since; of late very rapidly.

Has taken no medicine except a powder the 28th, which purged him violently, and a spermaceti mixture, his disorder

---

* Dr. M. has in this work pointed out with great accuracy, the dose of this poweful remedy, together with the most effectual mode of preparation and exhibition. He agrees with Darwin and others in prefering the infusion when the leaves of the plant have been recently dryed and preserved according to his directions.

In London practice, however, we think his tincture, which is kept at Apothecaries Hall, and by all the Chemists, a more certain preparation, as we cannot depend on the keeping of the leaves or powder in town, with the same confidence. The dose of the tincture is fifteen to thirty drops ter die. See our Journal, Vol. II. p. 123.

order having been considered as gout, to which he has been subject, both in a regular and irregular way, from eight years of age, and which appears to be hereditary. The gouty paroxysms have at times been very severe, and the leg now principally swelled has suffered the most. Has lived temperately. No cough.

Appl. empl. canth. pectori.

℞. Dig. purp. fol. p. nostr. gr. viij. Zing. p. gr. vi. Conf. arom. q. s. ft. boli viij. Sumt. 1. sing. noct. et mat.

℞. Bals. tolut. ℈ij. Muc. g. arab. ʒij. Mannæ ℥ß. tere simul. aq. menth. pip. ℥vjß. Sp. æth. vitr. c. sp. æth. nitrosi aa. ʒiij. M. sumt. coch. tria 6tis. horis.

April 4.—Immediate relief from the blister; and in the course of the following day, the 31st ult. the urine began to flow in increased quantity, but still high coloured with sediment, and he was able to lie down in bed. On the morning of the 1st, had five loose watery stools; but nevertheless the urine continued to increase considerably, and became perfectly natural in appearance: in the course of the day the swelling of the legs was nearly subsided; when it began to lessen, in the morning, he complained of very acute pains in them. Breathing now perfectly free; yet he still complains of some tightness across the chest. P. much more regular. Water since last night less in quantity, and deposits a sediment, but he has drank less. Countenance greatly improved; appetite perfectly restored; and he sleeps as usual in bed. No sensible effect from the digitalis except in increasing the flow of urine, and once on the bowels. The weather so intensely cold that he is prevented from going out and deriving advantage from air and exercise.

Appl. empl. e cymino inter scapulas, et empl. canth. alt. pect. si urget dyspnœa, nec non.

℞. Dig. p. p. gr. xij. Zing. p. gr. viij. Conf. arom. q. s. ft. boli viij. Sumt. j. mane et h. s. u. a.

Pergat in usu misturæ ter ind.

April 8.—Being alarmed by the appearance of unusual symptoms he desired my immediate attendance. He complained of being extremely faint and low, with giddiness and imperfect vision since yesterday. These were evidently the effects of the digitalis, having taken the last bolus this morning. In other respects was sensibly amended.

Omitt. dig. sed,

℞. Decoct. cinch. ang. ℥vij. Myrrhæ ʒj. Tinct. gent. c. ʒiij.

c. ℨiij. Sp. æth. nitr. ℨij. Kali pp. ℨß. M. sumt. coch. tria ter ind.

April 22.—Called on me to say that he continued in every respect free from dropsical symptoms; the legs being restored to their former state; the urine in full quantity; appetite good; and strength much improved, though the weather has been extremely unfavourable for his riding on horseback, as particularly enjoined. Both the plasters were applied, and the mixture persevered in since last report.

Was caught in a hail storm four days since, got wet, and has had some cough, with dyspnœa. P. irregular.

℞. Calom. gr. vi. Scillæ gr. iv. Digit. gr. x. Myrrhæ ℈j. Simul tere et adde g. asæfœt. ℨß. Extr. gent. q. s. ft. pilulæ xviij. Sumt. duas sing. noct. h. s.

June 8.—Again came to me. Says, that till a few days back he continued tolerably free from complaint; that his urine has diminished in quantity, with the usual sediment; that he has a slight cough, some difficulty in breathing, and a pain of the right side and breast. Looks well in the countenance, has no thirst; nor do the legs swell. Appetite and strength, however, somewhat impaired.

℞. Scillæ pulv. gr. x. Dig. purp. ℈j. Myrrhæ ℨj. Simul tere et confect. aromat. q. s. ft. pil. xxiv. Sumt. duas meridie et hor. viij. P. m. cum mist. seq. coch. iij.

℞. Infus. gent. c. ℥vij. Kali pp. ℨj. Sp. æth. vitr. c. sp. æth. nitr. a ℨiij. m. ft. mist.

Si urgeat dyspnœa iterum appl. empl. e pice burg. sterno.

The above symptoms were quickly removed, and he enjoyed his usual state of health till the autumn of 1800, when he again consulted me.

CASE XCIII.

June 17.—Early this morning my immediate attendance was desired on the Rev. Mr. ———, of ———, near Harwich. His age 51, of tall stature, naturally robust and muscular, but of late years disposed to corpulency. His symptoms were, extreme difficulty in respiration, which has for some weeks prevented him from lying down in bed, or from walking but with great deliberation: when going up stairs, has been obliged to rest some minutes to take breath, before he reached his chamber; and although propped up by pillows, nearly in a sitting posture, in bed, he is suddenly roused from sleep by a sense of suffocation, and extreme anxiety about the præcordia, which obliges him him to get up and walk about the room; of late this has happened three or four times every night, in so violent and alarming a degree, that his life has been despaired of; urine very scanty, of a dark red colour, but with very little sediment; pulse extremely irregular and intermitting; legs œdematous up to the knees, and readily pitting on pressure; tongue foul, with great thirst; no appetite; bodily strength much impaired; face and conjunctiva of a deep yellow tinge; and he brought up some yellow bile this morning. Bowels costive; memory much impaired, with giddiness, and confusion of ideas at times; constant drowsiness, amounting sometimes to stupor; hands and feet cold. Has not been addicted to intemperance in the use of intoxicating liquors, but in the habit of indulging very freely in large draughts of beer, both at meals, and at all times when heated by exercise. Has used much bodily exercise till of late years, when his habits have been sedentary and studious, frequently sitting up till a late hour.

For nine years has been subject to a peculiar uneasy sensation in the lower part of the abdomen, from the umbilicus to the pubis, for which he has been in the constant habit of using active purgatives three or four times a week, as the only means which, he fancied, relieved him.

About two years since, when stooping to measure timber, was suddenly seized with a giddiness of the head, and confusion of ideas, nearly amounting to a fit of apoplexy, for which he went to town, and put himself under the care of an eminent physician. It was treated as an attack of apoplexy, for which copious v. s. and cupping were frequently used. He returned to the country much reduced in flesh and strength.

The first symptom of his present complaint was perceived in February, 1806, when he was suddenly roused from sleep, and thought himself in the act of dying. He had another attack when doing duty, some months afterwards; and a third in December following: the pulse intermitted each time; recovered its former regularity after the first and second attacks, but has been more or less irregular since the third.

He has been attended by three physicians of the first eminence in town, and by one in the country. Their attention appears to have been principally directed to the affection of the head, and to the peculiar sensation of the abdomen, acknowledging they could do nothing for the disorder of the chest. The result of a consultation held by two two of them in town, in April last, was the recommendation of a seton in the neck, and mild inert palliative medicines. On the suggestion of Mr. Nunn, of Manningtree, the digitalis was once prescribed by one of them, but soon abandoned.

On the presumption that his complaints originated from water in the chest, Mr. Nunn and Mr. Silke his partner, who constantly attended, prescribed the digitalis in January and March last, with sensible advantage, as the pulse recovered its natural regularity both times: and Mr. Nunn reminding me of a case, apparently as hopeless as the present, in which I recommended the foxglove, with him, with complete success, lamented that the prejudice against it here was so strong, as to preclude him from prescribing it again.

On assuring the patient and his family, that the medicines I should recommend would produce no violent or unpleasant effects, they left me to proceed as I should judge fit, unfettered by any restrictions. Had a blister applied to the chest, and a calomel and squill bolus last night, which were the means of procuring some disturbed sleep.

℞. Calom. gr. vj. Kali vitr. myrrhæ aloes a. gr. x. Tere simul. et conf. ar. q. s. ft. pil. viij. quamp. sumat ij. et repr. p. r. n.

℞. Calom. gr. jß. Scillæ p. gr. j. Crys. tart. p. ℈j. Conf. ar. q. s. ft. bolus sing. noct. s.

℞. Inf. dig. p. (ʒjß. ad ℥viij.) ℥j. Sp. æth. nitr. ʒß. T. cort. aur. ʒj. Kali pp. gr. x. Ol. m. pip. gt. j. M. ft. haust. mane, mer. et vesp. per dies tres, vel iv. s.

He was directed to refrain from beer, but to drink freely of negus or gin-punch, acidulated with crystals of tartar; to have a few glasses of Madeira daily; and when his appetite returned, to have solid nutritive food, instead of the slops to which he had been accustomed. Mr. Nunn was requested not to continue the digitalis beyond the third day, if it should occasion the slightest nausea or unpleasant effect; and to substitute the infus. gent. c. for it; at all events not to persevere in it after the fourth day, having appointed to see him on the fifth.

June 22.—From voiding only half a pint of urine daily, for some time prior to my first visit, he passed, by measure, five pints every day after the first 24 hours, with proportionable relief of every symptom. For two days had been able to lie down in bed, and enjoyed sound uninterrupted refreshing sleep: swellings of the legs gone; appetite and strength returning; was able to walk without assistance sistance an hour and a half yesterday in his garden. Has had occasion to use the pills only once, the boluses having procured two motions daily without griping. No sickness or any unpleasant symptom from the foxglove. Took one draught to day with the infus. gent. c.

Pergat in usu haust. cum infus. gent. c. vice inf. fol. dig. repr. boli sing. noct. h. s.

June 29.—By previous appointment came twelve miles to meet me: has continued to mend; rides out every day in an open chaise, and drives himself; appetite craving, and he has indulged it too freely, especially at night, in consequence of which his stomach was disordered one night; and he has complained of giddiness and confusion of ideas at times.

Appl. empl. canth. nuchæ.

℞. Inf. gent. c. ℥jx. T. cort. aur. ℥j. Acidi nitr. gt. jv. ad. vj. m. ft. haust. cum pil. infra præs. No. ij. s.

℞. Myrrhæ, columb. p. a. ℈j. Zing. p. gr. x. Ol. m. pip. gt. jv. Syr. zing. q. s. ft. pil. xjv. Repr. boli u. a.

July 8.—Came seventeen miles to meet me, as previously agreed upon, and drove himself; continues to improve progressively; appetite more natural; and digestion good; head much relieved by the blister; has transacted business, and written letters, which required mental exertion. Walked up a hill yesterday a mile from home, and back again without inconvenience. Has recovered his natural florid complexion. Medicines to be continued, the draughts twice a day only.

July 22. Came as before to meet me. The day being excessively sultry, and being exposed for near four hours to an intense sun, I was surprised to find him very little affected by the heat. On the 13th he complained of great pain in the lower part of the abdomen, and in the course of the evening had three motions, consisting chiefly of dark grumous blood and a gelatinous like substance; the last without any fæculent matter. He mentioned with much satisfaction, that he has been since free from this painful complaint, which had more or less constantly harrassed him for nine years; and that for three nights the bowels performed their functions regularly, without any assistance. The gums being slightly affected, directed the boluses to be taken only occasionally. Having been formerly recommended the shower-bath, I permitted him to try it (especially as the weather had been intensely hot) every other morning. Acknowledges himself very much improved in every respect, and talks of making an excursion sion to Westmoreland in an open carriage. Was desired to go on as before.

It may be sufficient further to remark, that from this period Mr. H. either continued to meet me occasionally, or that I visited him at his own house, for some months. He became at length extremely impatient under the restraints imposed upon him; and notwithstanding every endeavour on the part of his amiable lady, the paroxysms of passion, when denied whatever he called for, were such as to render it impossible to keep him within bonnds; yet all that was possible that the most tender and affectionate regard could dictate, was done by her. She persevered to the last, and to the last the remedies did not fail to evacuate the water, and afford relief. His head, however, became more and more affected, and the bilious attacks more frequent and violent. He died on the 16th of July, 1808; yet the water was completely evacuated not many days before.

### CASE XCIV.

August 10, 1800.—Mr. Clover, a miller, æt. 53, of very short stature, thick make, and corpulent. Has worked hard, lived well, but not addicted to excesses in drinking. Has orthopnœa of the most distressing kind, even without any motion or exertion, being obliged frequently to raise his head suddenly, and to elevate his shoulders and arms at the same time, while he makes several laborious efforts to draw his breath; inability of lying down in bed for a fortnight, before which he could only lay on the right side. Is frequently roused from sleep suddenly, though he is supported by pillows in a sitting posture in bed. Urine high coloured, diminished in quantity, but without sediment; legs anasarcous to the knees, hard, resisting, and of a dark red colour; pulse full, and occasionally an intermission during the paroxysms of orthopnœa; face livid; no cough, pain of the chest, numbness of the shoulders, nor swelling of the abdomen; a peculiar oppression not only of the epigastrium but of the whole body; great debility and no appetite; much thirst. Disorder commenced abont December last, without any evident cause, and has been gradually increasing since. Has used various medicines without advantage, and was prescribed the following lately by a physician of extensive practice, who attended him.

℞. Fol. dig. p. ʒß. Aq. ferv. ℥xij. Infunde et ft. infus.

℞. Infus. colat. ℥j. Sp. æth. vitr. gt. xx. Tinct. opii c. ℈ij.

℈ij. Aq. ℥ij. Olei m. pip. gt. ß. M. ft. haust. 5tis. vel 6tis. horis s.

No sensible effect or relief was produced. I prescribed as follows.

℞. Infus. dig. p. (ʒiß. ad ℥viij.) ℥j. Sp. æth. nitr. ʒj. Kali pp. ℈j. M. ft. haust. ter ind. s.

Appl. empl. canth. pectori.

℞. Calom. scillæ a. gr. j. Opii gr. ß. Crystall. tart. p. gr. vj. Muc. g. Arab. q. s. ft. bolus sing. noct. h. s. s.

I consider the case in every respect as very unpromising.

August 15. In thirty-six hours after beginning the medicines, was sensibly relieved, and has continued to mend ever since, the urine flowing in full quantity, and the breathing and every other symptom being proportionably relieved; though he has taken his medicines very irregularly. Pergat, U. A.

Aug. 23. Improving in every respect, gaining strength, and able to walk out daily; lies down in bed, and breathes in every posture with freedom; more so in bed than in any other. Was directed to persevere in the same plan.

The water was repeatedly evacuated, and the symptoms were relieved by these medicines, but he relapsed soon afterwards, and at length laying aside all medicine, the disorder increased, and proved fatal, not many months after my first visit.

Though this case was omitted in its proper place, yet it is thought worthy of insertion here, not only as being in itself interesting, but as shewing the inert practice too generally adopted in this disease.

---

The combinations and formulæ most approved of by Dr. M. in his work, are,

1. The standard infusion, viz. ℞. Dig. purp. fol. incis. ʒiss. Canellæ alb. contus. vel zingib. incis ℈j. Aq. ferv. ℥viij. Infunde per horas iv. in vase operto; dein liquorem effunde.

If the leaves be used as they are suspended in paper bags in a room in which a fire is constantly kept in winter, without farther drying, which should always be done for infusion, the liquor will pour off perfectly clear, and save the trouble of straining. The stalks and large fibrous parts should be separated, and the leaves cut into small pieces.

2.—℞. Infus. dig. purp. ℥j. Aq. menth. pip. ʒiij. Kali pp. gr. x. ad ℈j. Sp. æth. nitrosi. vel sp. æth. vitr. c. ʒss. ad ʒj. M. ft. haustus bis terve de die sum.

3.—℞. Lin. camph. ℥ij. Sp. ammon. c. tinct. opii aa. ℥ß. M. ft. Lin. This

This liniment well rubbed upon the epigastrium or abdomen, I have repeatedly prescribed with immediate relief in violent sickness and vomiting, or acute pains of the bowels. The parts should be covered with hot flannel afterwards.

4.—℞. Dig. purp. fol. pulv. scillæ recent. exsicc. p. aa. gr. jx. Calom. gr. vj. Myrrhæ ℈ij. Crystal. tart. pulv. ℈j. Tere simul et extract. gent. q. s. ft. pil. xxiv. Sum. duas usque ad tres sing. noct. et mat. superbib. liquor. infra præs. cyathum vel libræ quadr.

5.—℞. Cryst. tart. pulv. ʒij. Solv. in aq. ferv. lib. j. add. rad. zing. incis. ʒß. Sacch. pur. q. s. Sp. juniper. ver. ℥iß. vel vini rhen. ℥iij. M. propinat æger quant hancce vel duplicem quotidie.

As soon as the patient begins medicine, the free use of this or some diluting drink, according to former habits, is directed as an absolutely necessary part of the plan.

6.—℞. Tinct. tolut. vel benz. c. ʒß. ad. ʒi. Muc. g. arab. vel ov. vit. q. s. ut miser. Infus. dig. purp. ℥i. Mell. scillæ ʒi. ad ʒij. Sp. æth. vitr. c. gtt. xxx ad ʒi. Kali pp. gr. x. ad ℈j. M. ft. haust. ter ind. s.

The author is not ignorant that here a kali acetat. may be formed, but the kali pp. will still predominate.

7.—℞. Fol. dig. purp. pulv. Scillæ pulv. aa. gr. i. Calomel. gr. ß. ad gr. j. Cryst. tart. ℈j. ad. ʒß. Syr. zingib. q. s. ft. bol. sing. noct. et mat. s. Besides this, No. 5 should be given freely.

8.—℞. Infus. gent. c. ʒix. Kali pp. gr. x. ad ℈j. Tinct. cort. aur. ʒiß. Sp. æth. nitr. gtt. 40 ad ʒi. M. ft. haust. ter ind. cum pil. infra præs. No. ij. s.

9.—℞. Ferr. vitr. kali pp. aa. ʒß. Myrrhæ ℈ij. Simul in pulv. subt. tere et extract. gent. q. s. ft. pil. xxiv.

10.—℞. Calomelan. gr. ij. ad. iv. Crystallor. tart. p. gr. x. Zing. pulv. gr. v. probe simul in pulv. subt. tere et syr. Zing. q. s. ft. bolus h. s. s. et seq. matutino degir. æger. haust. infra præs.

11.—℞. Infus. sennæ tart. vel inf. tamarind. cum senna (Ph. Ed.) ʒx. Mannæ ʒj. Tinct. sennæ vel jalap. ʒij. ad ʒß. M. ft. haust mane sumendus.

12.—℞. Tinct. benz. c. gtt. 40 ad 60 vel tinct. tolut. ʒi. Cum muc. g. arab. q. s. redact. Myrrhæ gr. x. Aq. m. pip. ʒi. æth. nitr. vel vitr. c. ʒi. Kali pp. gr. x. M. ft. haust. ter ind. s.

13.—℞. Resin. jalap. calomelan. zingiber. pulv. aa. gr. ij. ad iv. crystall. tart. pulv. gr. v. ad x. simul tere et syr. zing. q. s. ft. bolus h. s. s.

